# Study on the Process of Preparing Aluminum Foam Sandwich Panel Precursor by Friction Stir Welding

**DOI:** 10.3390/ma17204981

**Published:** 2024-10-11

**Authors:** Yu Zhang, Qiu Pang

**Affiliations:** 1 Hubei Longzhong Laboratory, Wuhan University of Technology, Xiangyang 441000, China; yuzhangzyf@163.com; 2 Hubei Key Laboratory of Advanced Technology for Automotive Components, Wuhan University of Technology, Wuhan 430070, China; 3 Hubei Research Center for New Energy & Intelligent Connected Vehicle, Wuhan University of Technology, Wuhan 430070, China; 4 Department of Mechanical and Electrical Engineering, Wuhan Donghu University, Wuhan 430212, China

**Keywords:** friction stir welding, process parameters, aluminum foam sandwich, numerical simulation

## Abstract

In recent years, high-performance lightweight and multifunctional aluminum foam sandwiches (AFSs) can be successfully applied to spacecraft, automobiles, and high-speed trains. Friction stir welding (FSW) has been proposed as a new method for the preparation of AFS precursors in order to improve the cost-effectiveness and productivity of the preparation of AFS. In this study, the AFS precursors were prepared using the FSW process. The distribution of foaming agents in the AFS precursors and the structure and morphology of AFS were observed using optical microscopy (OM), scanning electron microscopy (SEM), and X-ray energy dispersive spectroscopy (EDS). The effects of the temperature and material flow on the distribution of the foaming agent during the FSW process were analyzed through experimental study and numerical simulation using ANSYS Fluent 19.0 software. The results show that the uniform distribution of the foaming agent in the matrix and excellent densification of AFS precursor can be prepared when the rotation speed is 1500 r/min, the travel speed is 25 mm/min, the tool plunge depth is 0.2 mm, and the tool moves along the retreating side (RS). In addition, the experimental and numerical simulations show that increasing the welding temperature improves the uniformity of foaming agent distribution and the area of AFS precursor prepared by single welding, shortening the thread length inhibits the foaming agent from reaching the upper sandwich plate and moving along the RS leads to a more uniform distribution of the foaming agent. Finally, the AFS with porosity of 74.55%, roundness of 0.97, and average pore diameter of 1.192 mm is prepared.

## 1. Introduction

Aluminum foam is widely used in many industrial fields due to its remarkable properties, such as low density, high specific strength, sound insulation, and energy absorption [1,2]. However, the mechanical properties and surface flatness of aluminum foam are unfavorable; they are easy to corrode and have other shortcomings, which limits their further applications [3]. Aluminum foam sandwich panels (AFS) are manufactured by a special processing method that covers the panels in the outer layer of aluminum foam to produce an aluminum foam composite structure (AFCS), which not only retains the excellent performance of the core layer of aluminum foam but also improves the load carrying capacity of the aluminum foam [4]. AFS panels are widely used in the military, automotive, shipbuilding, aerospace industries, and railroad industries due to their high specific stiffness and strength, excellent specific energy absorption performance, and having the characteristics of low density [5,6].

At present, the preparation methods of AFS panels are divided into adhesive connection method, powder metallurgy method, melt foaming method and welding method [4]. The adhesive connection method can face problems such as melting at high temperatures, easy deterioration under corrosive conditions, and aging in use [7,8]. The powder metallurgy method is usually combined with a pressing process or a rolling process, which requires a certain pressure and temperature to make an AFS panel precursor [9]. The combination of powder metallurgy and rolling process can improve the density and interfacial bonding strength of the core powder, but the phenomenon of powder aggregation and powder missing can easily occur [4,10]. Due to the limitations of the manufacturing equipment and the complicated production process, it cannot satisfy mass production in industry [11]. Melt foaming not only requires the transfer of the molten aluminum matrix but also the bubbles float upwards under gravity, which leads to difficulties in controlling the size uniformity and distribution uniformity of the bubbles [7,12].

Welding methods to prepare foam aluminum sandwich panels are mainly divided into brazing and friction stir welding (FSW). Brazing uses a low melting point of the brazing material to achieve the joint of the AFS panel. However, the appropriate filler metal, the oxide film on the surface of the aluminum foam, the welding time, and temperature have a great influence on the strength of the joints, which are prone to contain brittle intermetallic formation and low-temperature eutectic constituents at the joints [12,13,14,15]. FSW is a new and highly efficient solid-state joining technology [16]. The preparation of AFS precursors by FSW avoids the problems of high cost, powder agglomeration, and difficult density control in the traditional process. There are three main methods to prepare AFS panels by FSW: (1) Peng et al. [17] successfully prepared AFS panels by welding the panels to the aluminum foam by FSW. (2) Su et al. [18] combined the FSW process with the rolling process to weld the AFS precursors obtained by rolling. After insulation, interfacial metallurgical bonding is achieved between the unconnected precursors. (3) AFS precursor is prepared by adding a foaming agent to an aluminum plate by FSW process, and then after holding in a heating stove to obtain an AFS panel [19,20]. This method can effectively prevent the poor compactness of the AFS precursor and the accumulation of the foaming agent, and it can realize the metallurgical bonding of the foam core and panel after insulation.

Multi-pass FSW can prepare the large-area AFS precursor by varying the insertion position of the tool and its motion relative to the workpiece. In multi-pass FSW, the quality of the weld is influenced by the welding parameters and the tool profile [16,21,22]. Welding parameters for the preparation of AFS panel precursors by FSW have been studied by many scholars. Pang et al. [23,24] investigated that when a 3 mm aluminum alloy plate was welded at a travel speed of 50 mm/min and rotation speed of 2000 rpm, the material in the weld zone (WZ) experienced enough plastic deformation and flow. Hasegawa, M. [25,26] et al. showed that uniform mixing of TiH_2_ and Al_2_O_3_ powders inside the precursor could be achieved at a travel speed of 30 mm/min at over 2200 rpm. Kohutiar et al. [27] studied the influence of workpiece geometry on welding quality and mechanical properties. By comparing three geometric shapes, they obtained the optimal welding quality geometry and analyzed the reasons behind the influence of geometric shapes. Muhayat et al. [28] investigated the joint mechanical properties and weld microstructure characteristics of three different tool pin profiles. Tunnel defects were found in the welding area of cylindrical and double-plane pin shapes, while the mechanical properties of three-plane pin shapes were the best. Heat generation in FSW depends on the friction between the tool and the workpiece. The geometry of the tool significantly controls the heat generation, along with process parameters such as rotational speed and traverse speed. The pin controls the movement of plasticized materials during welding [29,30]. Consequently, the design of both the shoulder and the pin is crucial for achieving sound welding.

During FSW, material flow has a significant impact on the foaming agent distribution in the WZ. However, experimental methods make it difficult to characterize the material flow around the tool and cannot deeply clarify the potential mechanisms. Therefore, numerical simulation methods are needed to assist in solving this problem. The computational fluid dynamics (CFD) method has been widely used in the simulation of FSW processes due to its ability to solve complex nonlinear material flows caused by large deformations [31,32]. Chen, G. et al. [33] used a numerical simulation method based on Computational Fluid Dynamics (CFD) to analyze the effect of pin threads on material flow during FSW of an Al–Mg–Zn alloy. The effect of pin threads on material flow was elucidated. Kadian, A. K. et al. [34] investigated the material flow motion throughout the WZ and proposed a new material flow model for FSW of dissimilar materials. The effects of tool speed and welding speed on the material motion were obtained. Yang, C. et al. [35] predicted the temperature field and material flow field near the tooling interface during the FSW process based on experimental studies and three-dimensional computational fluid dynamics (CFD) analyses, which were verified by measured temperatures and weld macroscopic maps.

Based on the above studies, factors such as welding parameters, tool profiles, and multi-pass welding methods are important for the distribution of foaming agents in the AFS precursor. Therefore, in this study, the effects of different welding parameters, tool profile and the direction of tool travel movement on the organization and morphology of the AFS precursor and the quality and position of powder mixing are investigated. A three-dimensional numerical simulation based on the CFD method is established to analyze the temperature field and the material flow field in the FSW process. On the basis of numerical simulations, the influence of pin threads on the material flow behavior during the preparation of AFS precursors is elucidated, and the influence factors of foaming agent powder distribution are revealed.

## 2. Experimental Procedures

**Figure 1:**
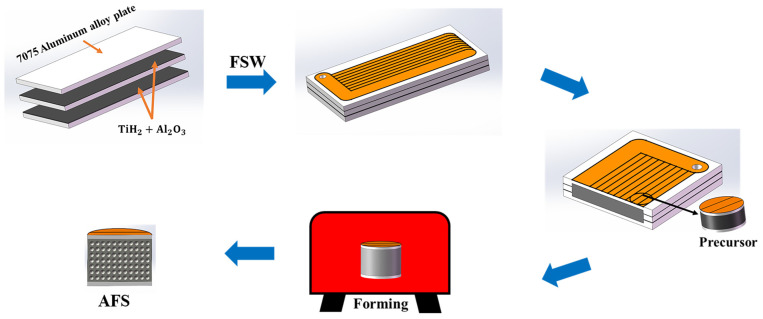
Schematic diagram of the process of preparing AFS by FSW.

The 7075-O aluminum alloy (Al-Zn-Mg-Cu) sheet (250 mm × 120 mm × 2 mm) was selected as the base material, and the mechanical properties are shown in Table 1. Due to its good plasticity and molding properties, it can effectively avoid defects such as cracks, tunnel defects, and excessive weld flash during the welding process. TiH_2_ powder (~45 μm) and Al_2_O_3_ powder (~1 μm) were mixed for 120 min as the powder using a planetary ball mill. FSW tools were made of H13 tool steel and designed with threaded pin diameters of 5.5 mm with different thread lengths to prepare AFS panel precursors. The foaming agent was uniformly placed between three plates. The multi-pass welding was performed on the model NFSW-650 FSW machine developed by the Shenyang Institute of Automation of the Chinese Academy of Sciences (Shenyang, China). A tool plunge depth of 0.2 mm was used in this experiment. The temperature distribution during the welding process was tested by the FOTRIC-226 intelligent thermal imaging camera developed by the Shanghai FOTRIC Technology Company (Shanghai, China). The schematic diagram of the process of preparing the AFS structure by FSW is shown in Figure 1.

After the metallographic specimens of AFS precursors were corroded using Keller’s reagent (HNO_3_:HCl:HF:H_2_O = 2.5:1.5:1:1:95), the microstructural characteristics of the AFS precursors were observed by optical microscopy (OM) and scanning electron microscopy (SEM). The distribution of the foaming agent in the aluminum plate was observed by spot scanning and surface scanning of the important elements Ti, Al, and O using EDS. Metallographic specimens were prepared after foaming of the AFS precursor, and the microstructural characteristics of the foams were observed under an optical metallographic microscope. The average equivalent diameter (d) and average circularity (e) of the pores were calculated using the area and perimeter of the pores calculated by Image-Pro Plus 6.0 software. The equivalent diameter (d) and circularity (e) of a pore were evaluated as

(1) $$d=2{\left(\frac{A}{\pi }\right)}^{0.5}$$

(2) $$e=\frac{4\pi A}{L^{2}}$$

where *A* is the pore area, and *L* is the pore perimeter. *A* value of circularity closer to 1 indicates a more circular pore.

## 3. Mathematical Modeling

**Figure 2:**
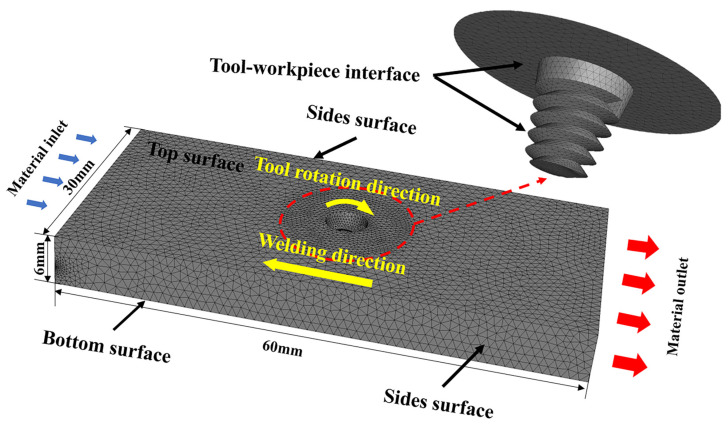
Geometry, boundary definition, model mesh of the numerical model.

The large-scale commercial fluid dynamics (computational fluid dynamics (CFD)) software ANSYS Fluent 19.0 is used to solve the developed model. In the mathematical modeling, the effect of the inclination angle of the tool is not considered. The thermoplastic material near the pin is assumed to be a non-Newtonian, incompressible, viscoplastic material. The flow state is laminar in the whole calculation area. The flow stress and viscosity of the material are considered as a function of temperature and strain rate only. The pin is assumed to be rigid and not included in the model, and only the pin–workpiece contact interface is treated as a wall [37,38]. The coordinate origin is located at the intersection of the pin axis line, and the lower surface, and the size of the model computational domain is 60 mm × 30 mm × 6 mm. Mesh division is made in the ANSYS Workbench Meshing; the computational area is meshed using a non-uniform tetrahedral structure mesh with a minimum mesh size of 0.2 mm. Due to the large flow velocity and temperature gradient of the plastic material near the mixing head, a dense, fine mesh is used, while the temperature gradient and material flow velocity are relatively small in the area away from the mixing head and a relatively coarse mesh phase is used. The mesh division is shown in Figure 2.

During the welding process, the material in the vicinity of the pin is at a high temperature, high strain state, away from the pin of the base material area of low temperature and material deformation can be basically ignored. The distinction between the different regions is achieved by means of the Sellars–Tegart/Sheppard–Wright intrinsic model, and the equations for the viscosity, flow stress, and effective strain rate of the material are obtained according to Ji, H. et al. [32]. In the simulation of the FSW process, the heat generation model and boundary conditions, reproduced from Shi, L. et al. [39], where the material parameters of the workpiece at different temperatures are shown in Table 2. The model in this study uses a pressure-based detached solver to solve the conservation equations, and the algorithm is the Semi-Implicit Method for Pressure-Linked Equations Consistent (SIMPLEC) [38]. The material physical parameters and boundary conditions in the calculation are programmed and defined in C. The secondary development of ANSYS Fluent 19.0 software is carried out through the User Defined Function (UDF) module to realize the simulation of the FSW welding process.

## 4. Results

### 4.1. FSW Welding Parameters

**Figure 3:**
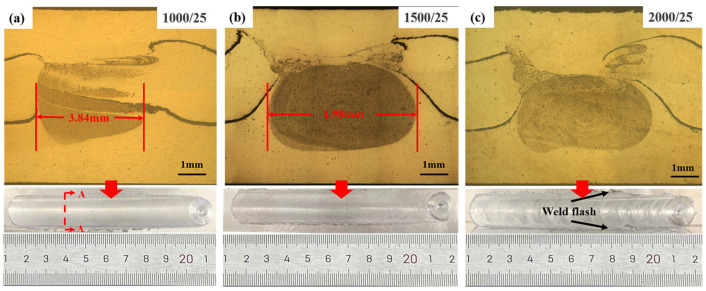
Comparison of the metallographic organization of AFS precursors at the travel speed of 25 mm/min and different rotation speeds: (**a**) 1000 r/min, (**b**) 1500 r/min, (**c**) 2000 r/min.

Figure 3 shows the metallographic organization of the AFS precursor at different rotational speeds of the tool at the travel speed of 25 mm/min. The letters A-A indicate the cross-sectional morphology observation intercept position. From the surface morphology of the weld in Figure 3, it can be seen that the surface quality of the weld first decreases and then increases with the increase of rotational speed. At a rotational speed of 1000 r/min, the region containing the foaming agent is not only smaller, but the distribution of the foaming agent powder is not uniform. When the rotational speed is increased to 1500 r/min, the powder is uniformly distributed in the middle layer of the weld area. The width of the AFS precursor for each FSW preparation is 1.14 mm larger than at 1000 r/min. The width of the AFS precursor prepared by each FSW is about 0.5 mm wider than the pin diameter. Although the same powder distribution effect can be achieved in the 2000 r/min and 1500 r/min welding areas, the low surface quality and larger weld flash at 2000 r/min will affect the subsequent multi-pass FSW.

**Figure 4:**
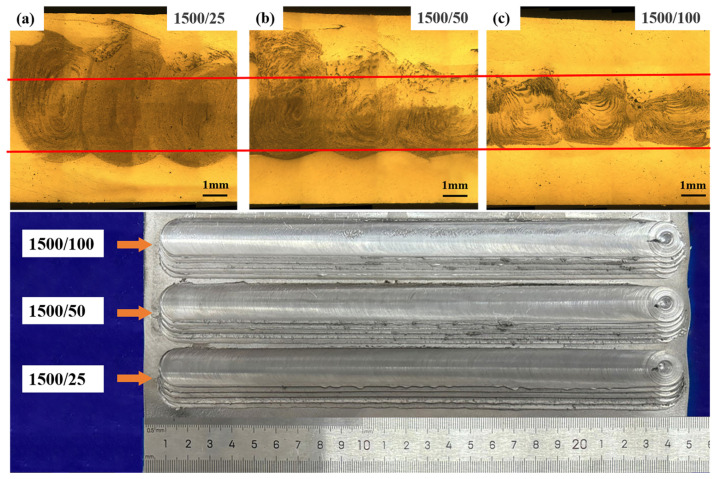
Comparison of metallographic organization of AFS precursor at the rotation speed of 25 mm/min and different travel speeds: (**a**) 25 mm/min, (**b**) 50 mm/min, (**c**) 100 mm/min.

Figure 4 shows the metallographic organization of the AFS precursor at different travel speeds at a rotation speed of 1500 r/min. At the travel speed of 25 mm/min, the powder has the structure of an onion ring, which is uniformly distributed in the WZ. The travel speed is increased to 100 mm/min, the structure of the onion rings in the WZ is changed, the gap between each ring increases, and the powder distribution area is small and inhomogeneous. With the increase in travel speeds, the surface quality of the weld is unchanged, but the uniformity of the foaming agent in the precursor is poor, as shown in Figure 4.

**Figure 5:**
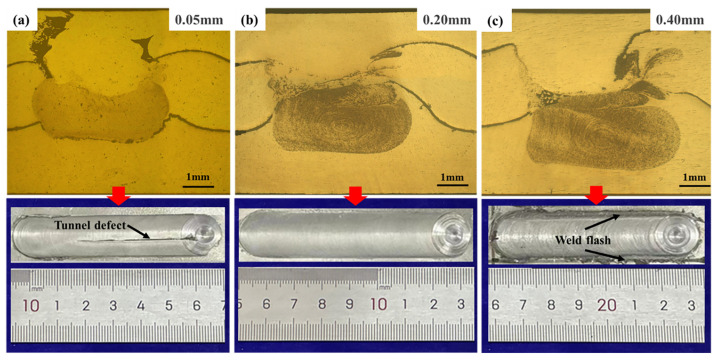
Comparison of metallographic organization of AFS precursors at different tool plunge depths: (**a**) 0.05 mm, (**b**) 0.20 mm, (**c**) 0.40 mm.

Figure 5 shows the metallographic organization of the AFS precursor under different tool plunge depths at the rotation speed of 1500 r/min and the travel speed of 25 mm/min. The tool plunge depth can seriously affect the quality of the weld, as shown in Figure 5. The small tool plunge depth will seriously reduce the extrusion effect of the shoulder and generate less heat, resulting in poor material fluidity and the inability to completely fill the cavity. The cracks and holes appear on the weld surface, seriously affecting the mechanical properties of the AFS precursor. The excessive tool plunge depth causes the surface quality of the weld to be reduced, with larger weld flash on both sides, making it easy for cracks to appear in subsequent multi-pass welding processes. At the same time, it causes serious panel thinning, with different panel thicknesses on both sides. At the tool plunge depth of 0.2 mm, the AFS precursor prepared by FSW is free from any defects, as shown in Figure 5b.

### 4.2. FSW Tool Movement Direction

**Figure 6:**
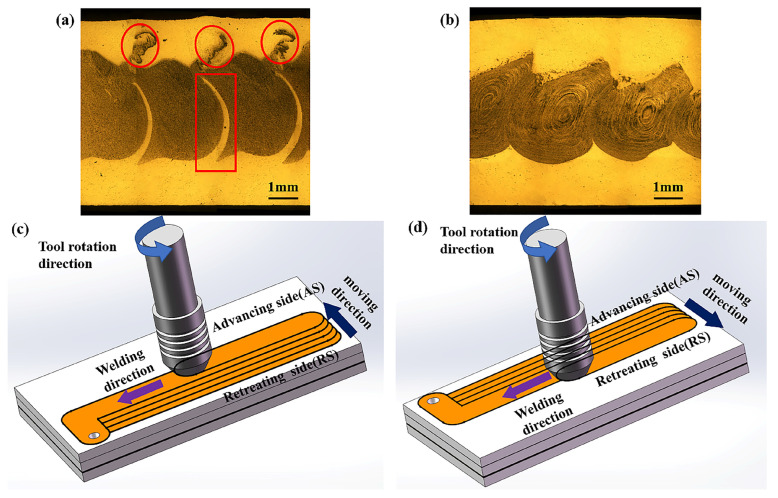
The schematic diagram of tool movement along different directions and comparison of metallography: (**a**) the metallography of AS, (**b**) the metallography of RS, (**c**) the schematic diagram of AS, (**d**) the schematic diagram of RS.

It is well known that during FSW, the tool produces a different material flow on the advancing side (AS) and retreating side (RS), thus creating asymmetric weld characteristics in the WZ [37]. In multi-pass welding, due to the asymmetry of material flow on both sides of the pin, the tool moves in different directions, and the foaming agent is distributed in different areas of the AFS precursor, as shown in Figure 6. As the tool moves along the AS, the powder mixing zone formed by the two welds does not fit tightly, with a substrate area in the middle devoid of powder, as indicated by the red box in Figure 6a. At the same time, internal crack defects appeared in the upper half of the RS of each weld, as shown by the red circle in Figure 6a. When each weld moves along the RS, there are no internal defects, the AFS precursor is a sandwich structure, the powder is evenly distributed in the middle of the plate cross-section, and the thickness of the sandwich is basically the same on both sides, as shown in Figure 6b. Therefore, the welding parameters for the preparation of AFS precursors by FSW have been selected, as shown in Table 3.

**Figure 7:**
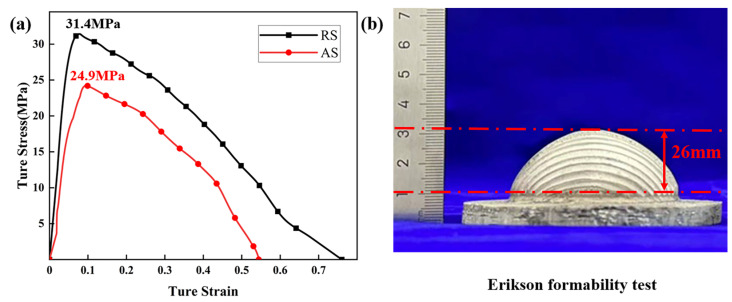
The formability experiment of AFS precursor under different welding tracks: (**a**) true stress-strain curves at different welding track, (**b**) Erikson formability test.

Figure 7 shows the formability experiment of AFS precursor under different welding tracks. Due to the inferior mechanical properties of AFS precursor at room temperature, it is impossible to prepare complex surface AFS. The formability of the AFS precursor increases first and then decreases with the increase in temperature, and the formability is the best at 400 °C [24]. The precursors were prepared through 17 pass FSW at a tool rotation rate of 1500 rpm, a travel speed of 25 mm/min and the tool moves along the RS. The tensile strength can reach 31.4 MPa, and the elongation is 76.02%. However, when the tool moves along the AS, the tensile strength is only 24.9 MPa, and the elongation is only 54.43% due to the uneven internal powder mixing and the occurrence of defects, as shown in Figure 7a. The Erikson formability test was carried out on the large-area AFS precursor prepared by multi-pass welding along the RS. The maximum cupping test values of the precursor could reach 26 mm, which is similar to that of the 7075-O aluminum alloy rolled sheet, as shown in Figure 7b.

### 4.3. FSW Tool Geometrical Features

**Figure 8:**
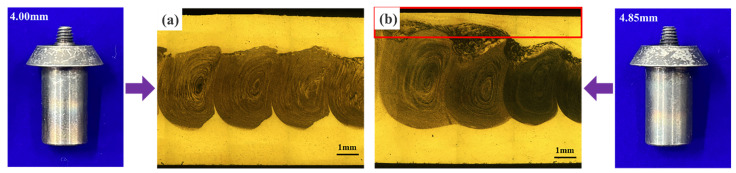
Comparison of metallographic organization of AFS precursors at different pin thread lengths: (**a**) 4.00 mm, (**b**) 4.85 mm.

The tool profile has a great influence on the material flow during the FSW process [21], and whether the foaming agent is uniformly distributed in the aluminum matrix is closely related to the material flow in the FSW caused by the pin profile. Figure 6 shows the comparison of the metallographic organization of precursors prepared with the same pin size with different pin thread lengths. When the pin thread length is 4.0 mm, the foaming agent in the prepared AFS precursor is uniformly distributed in the middle core layer, as shown in Figure 8a. When the pin thread length is 4.85 mm, the prepared AFS precursor contains a foaming agent in the upper sandwich layers, which is no longer a sandwich structure, as shown in Figure 8b.

## 5. Discussion

### 5.1. Effect of Welding Temperature on AFS Precursors

**Figure 9:**
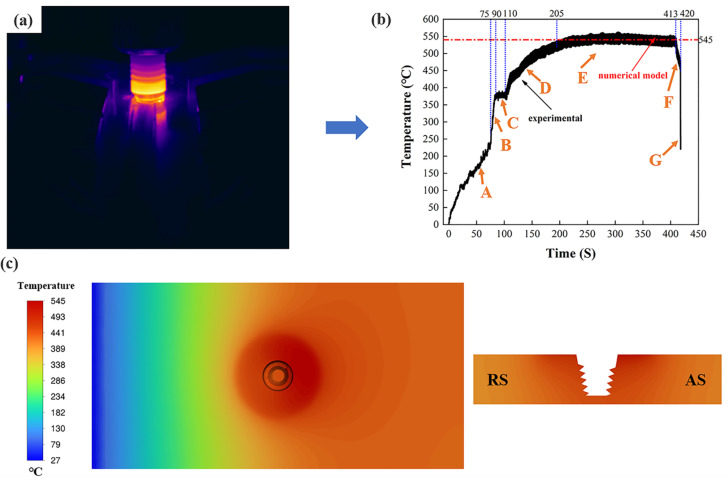
Comparison of experimentally measured and numerically calculated temperature at 1500 r/min rotational speed and 25 mm/min travel speed: (**a**) experimentally measured schematic, (**b**) experimentally measured temperature curve, (**c**) numerically calculated temperature.

Figure 9 shows the comparison between the experimentally measured and numerically calculated temperatures at 1500 r/min rotational speed and 25 mm/min travel speed. In stage A, only the friction between the pin and the material and the material flow driven by it produces heat, so the weld temperature only rises at a rate of 3 °C/s. In stage B, the shoulder contacts the upper surface of the plate, and the friction surface between the pin and the surface of the workpiece increases, so the welding temperature rises at a rate of 10 °C/s. In stage D, the heat of large plastic deformation of the metal in the internal shear layer of the base material raises the welding temperature again. Thus, the heat input required for welding comes mainly from the frictional heat generated between the tool surface and the base metal and from the heat of large plastic deformation of the metal. When the pin has traveled a certain distance, the welding temperature reaches a relatively stable state, as shown in Figure 9, stage E. When the weld reaches the steady state phase, the experimental results have temperatures ranging from 530 °C to 550 °C, and the numerical calculations have temperatures of 545 °C. The experimental results are in good agreement with the numerical simulations, indicating that the model can be used to predict the temperature distributions and material flow.

The material flow rate in the AS is the same as that in the travel direction. The plastic material flow rate in the AS is greater than that in the RS. The heat of large plastic deformation of the metal causes the overall temperature in the AS to be slightly higher than that in the RS. The temperature distribution in the WZ shows a distinctly asymmetric hourglass shape in the direction along the thickness; the area that can reach the softening of the matrix metal is larger on the AS than on the RS, and the powder mixing region has a larger distribution area on the AS than on the RS. When the tool is moved along the AS, the small stirring zone on the RS is not able to stir the powder layer completely between the two adjacent WZ and crack defects containing powder appear in the core layer of the AFS precursor, as shown in Figure 6a.

**Figure 10:**
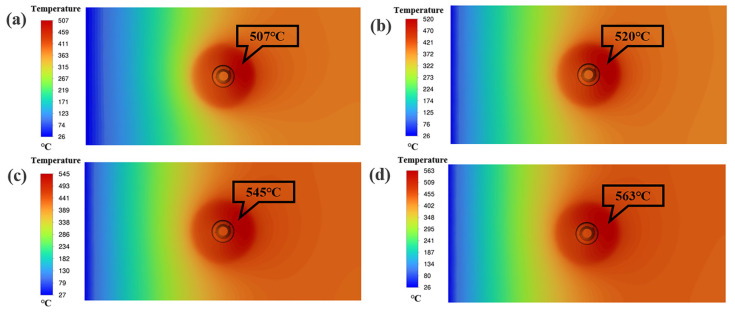
Numerical simulation of the temperature distribution on the top surface of the workpiece for different parameters: (**a**) 25 mm/min travel speed and 1000 r/min rotational speed, (**b**) 100 mm/min travel speed and 1500 r/min rotational speed, (**c**) 25 mm/min travel speed and 1500 r/min rotational speed, (**d**) 25 mm/min travel speed and 2000 r/min rotational speed.

**Figure 11:**
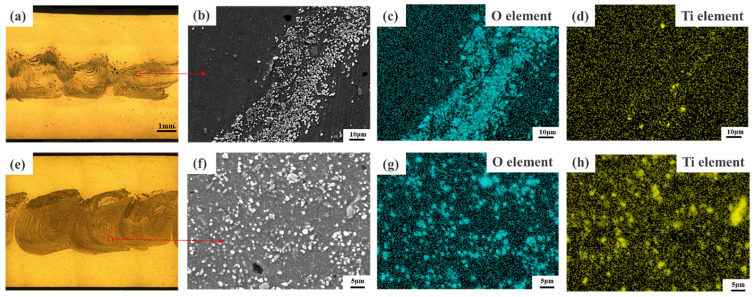
The SEM morphology and EDS analysis of precursors at rotational speed 1500 r/min and tool movement along RS: (**a**) the metallographic organization of travel speed 100 mm/min, (**b**) SEM image of the rectangular region in (**a**), (**c**,**d**) the EDS maps corresponding to (**b**), (**e**) the metallographic organization of travel speed 25 mm/min, (**f**) SEM image of the rectangular region in (**e**), (**g**,**h**) the EDS maps corresponding to (**f**).

Figure 10 shows the cloud diagram of temperature distribution on the top surface of the workpiece for different parameters. Due to the working temperature of 507 °C for 25 mm/min travel speed and rotational speed of 1000 r/min, the material temperature in the WZ is low, the fluidity of the metal is poor, and the flow rate of the material is low for this parameter. As a result, the distribution area of the foaming agent in the sheet is small and not uniform, as shown in Figure 3a. As shown in Figure 11e,f, the welding temperature is 545 °C under the parameters of 25 mm/min travel speed and 1500 r/min rotational speed. The prepared AFS precursor did not show any defects, and the powder was uniformly distributed in the core layer. In the EDS maps, Ti and O elements are uniformly distributed in the WZ, indicating that the TiH_2_ and Al_2_O_3_ are uniformly distributed in the matrix, as shown in Figure 11g,h. As the welding temperature increases, the area within the cross-section that can reach the softening temperature of the plate increases, which means that the WZ increases in a single pass. Therefore, the width of the AFS precursor obtained at 1000 r/min is smaller than that obtained at 1500 r/min.

At a travel speed of 25 mm/min and a rotational speed of 2000 r/min, the working temperature is 563 °C. Excessive temperatures result in a weakening of the deformation resistance of the metal in the WZ, while the deformation resistance of the materials farther from the center of the weld is still stronger. The plasticized metal receives extrusion from the shoulder, and the base material, and part of it overflows outward along the edge of the shoulder to produce a flash, as shown in Figure 3c. Under the parameter of travel speed of 100 mm/min and a rotational speed of 1500 r/min, the welding temperature ranges from 520 °C, the low temperature leads to low softening of the material. The distribution area of the foaming agent in the plate is small and not uniform due to the reduced area within the cross-section that can reach the softening temperature of the plate and the poor fluidity of the material, and the O and Ti elements are clustered on the onion rings, as shown in Figure 11a–d.

### 5.2. Effect of Material Flow on AFS Precursors

**Figure 12:**
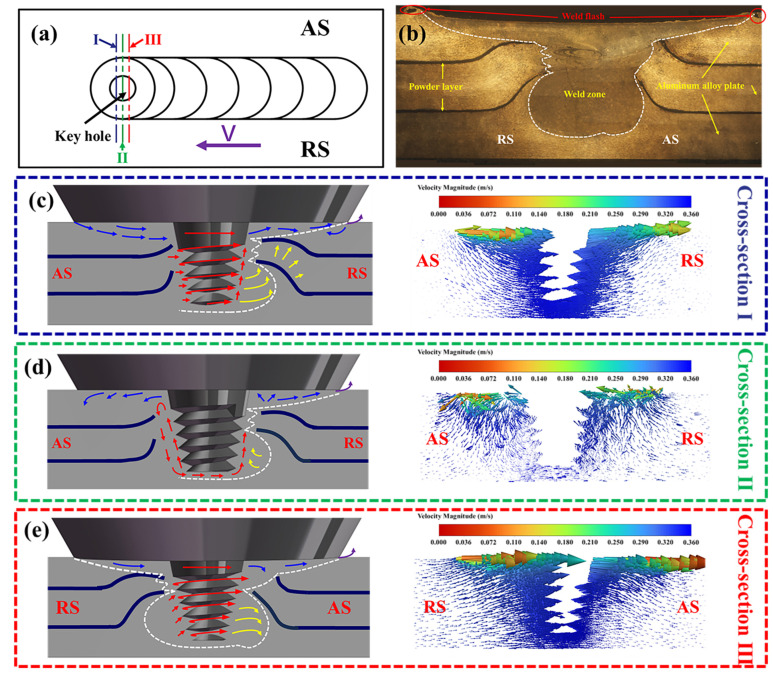
Material flow in cross-section at different locations around the pin: (**a**) the cross-section schematic at different locations around the pin, (**b**) the metallographic organization of WZ, (**c**) the weld metal flow of cross-section I, (**d**) the weld metal flow of cross-section II, (**e**) the weld metal flow of cross-section III. The red arrows represent the material flow caused by the pin, the blue arrows represent the material flow caused by the tool shoulder, the yellow arrows represent the material flow caused by the material buildup, and the purple arrow represents material flowing out of the shoulder to form weld flash.

**Figure 13:**
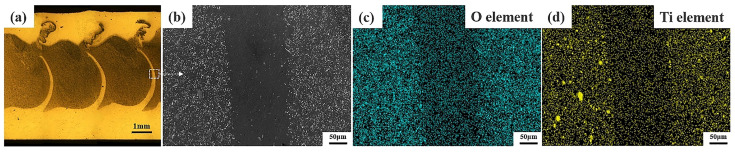
The SEM morphology and EDS analysis of AFS precursor welded along the AS at travel speed 25 mm/min and rotational speed 1500 r/min: (**a**) metallographic organization, (**b**) SEM image of the rectangular region in (**a**), (**c**,**d**) the EDS maps corresponding to (**b**).

On the AS and RS of FSW, the material flow and stress distribution are completely different. Figure 12 shows the material flow in cross-section at different locations around the pin. The velocity vector diagrams of cross-sections I, II, and III shown in Figure 12a are selected to study the plastic flow of weld metal. In cross-section I, the plasticized material flows from the AS to the RS. The material on the AS away from the pin flows downward first by the action of the shaft shoulder and the surrounding material, but with the low temperature of the base material at the bottom and the restriction of the plate, most of the material is carried by the pin to the RS. The plasticized material shows an upward flow phenomenon due to the constraint of the unplasticized base material on the RS, as shown in Figure 12c. At the same time, the deformation of the material at AS and RS is asymmetrical; the plastic strain at AS is higher than that at RS, and this plastic deformation increases to improve the material flow [40]. In addition, due to more heat generated on the forward side and greater material flow, the residual stress on the forward side is higher than that on the backward side [41,42]. Therefore, welding along the front side is prone to defects, as shown in Figure 13a. There is an upward movement of the powder interlayer on the RS of the weld, as shown in Figure 3. When the tool is moved along the AS, the small WZ on the RS results in an unstirred zone between two adjacent WZ. As material builds up on the RS, the matrix in the unstirred zone is extruded into a crescent shape, while the powder interlayer is pushed upwards to form cracks, and the WZ of the last weld flows upward, as shown in Figure 13a,b. In the EDS maps of Figure 13b, in the blank region between the two adjacent WZ, the content of O and Ti elements is extremely low, indicating that this region is the matrix, as shown in Figure 13c,d.

In cross-section II, the material on the AS moves upward along the threads of the pin to the shoulder, and then part of it flows toward the RS with the rotation of the shoulder, and the other part is squeezed and flows downward. The softened material appears to build up as it reaches the RS, further aggravating the upward flow of material on the RS, as shown in Figure 12d. Under the joint action of the shoulder and the threaded pin, the majority of the material flows to the backward cavity with the rotation of the shoulder, while a small part of the material flows out of the shoulder to form a flash, as shown in Figure 12b. As the material flows upward with the threads of the pin, the foaming agent powder is brought to the upper surface with the threads, and neither the AFS precursor nor the AFS is a sandwich structure, as shown in Figure 8b. When there is no thread at the root of the pin after the material flows out of the threaded region with the thread upward, the vertical pressure gradient on the material is reduced [43], which can effectively alleviate the powder from being carried to the upper surface, and its prepared AFS precursor and AFS are both sandwich structures as shown in Figure 7a.

In cross-section III behind the pin shown in Figure 12e, the material on the RS moves upward under the action of the thread. The material flows from the RS to the AS under the action of the shoulder extrusion and the rotation of the pin. At the same time, the cavity brought about by the movement of the pin is filled with most of the plasticized material in the process, and a small portion of the material flows to the AS with the rotation. The shoulder of the shaft drives the material flow more than the lower pin. The part near the shoulder is filled first, and then the cavity is completely filled by the excess material flowing down and the material brought by the rotation of the pin, as shown in Figure 12d. Meanwhile, less material reaches the AS in this process than the material reaches the RS at section I. After the material reaches the AS, the softened material appears to flow downwards under the influence of the shaft shoulder and the base material. The material on the RS is subjected to much less upward compression than on the AS. As a result, the tool moves along the RS can cause a very small rise in the foaming agent-containing zone and no blank area in the middle of each weld, as shown in Figure 6b.

### 5.3. AFS Precursors

**Figure 14:**
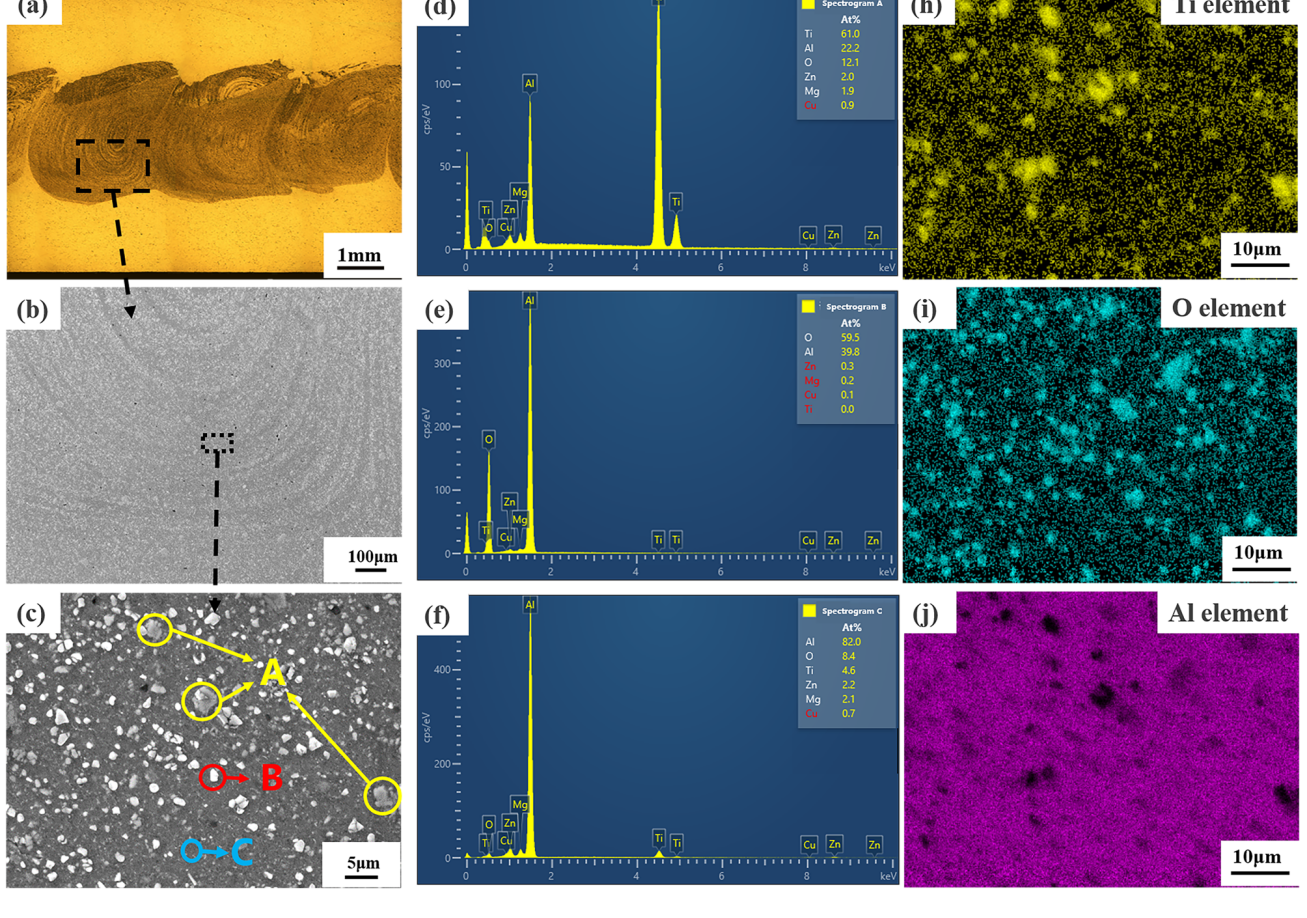
SEM morphology and EDS analysis of AFS precursor under optimal welding parameters and methods (**a**) metallic organization of the precursor; (**b**) SEM image of the rectangular region in (**a**); (**c**) enlarged image of the rectangular region in (**b**); (**d**) EDS spectra of region A in (**c**); (**e**) EDS spectra of region B in (**c**); (**f**) EDS spectra of region C in (**c**); (**h**–**j**) EDS maps corresponding to (**c**).

Figure 14 shows the SEM morphology and EDS analysis of the AFS precursor mixing region at the optimal welding parameters. Within the mixing area, most of the area is the same as the gray-black area indicated by C in Figure 14c; most of the white particles are covered by a layer of gray color as the area indicated by A in Figure 14c, and a few areas are the same as the area of bright white particles indicated by B in Figure 14c. In the EDS point-scan spectrum of the three regions A, B, and C, the content of the Ti element is 61% in region A. In region B, the ratio of the content of Al element to O element is 2:3, and the content of other elements is very low. The C region has the highest content of elemental Al, with elemental O and Ti ranking second and third, respectively. Thus, the bright white particles in the mixed region of the AFS precursor are Al_2_O_3_, the gray–black region is the Al matrix, and the TiH_2_ powder is distributed in the precursor as a gray cloud. The elements of Ti and O in the AFS precursor are scanned by EDS. The element O is uniformly distributed throughout the mixing zone and corresponds to the white particles, and the element Ti is almost uniformly distributed in the precursor, as shown in Figure 14h,i. Therefore, AFS precursors with uniform mixing of TiH_2_ and Al_2_O_3_ in the Al matrix can be obtained with the welding parameters in Table 3. Meanwhile, the TiH_2_ powder is distributed around Al_2_O_3_, which can ensure that the viscosity of the Al matrix around the bubble holes increases during the heat insulation process and enhances the roundness and uniformity of AFS bubbles.

**Figure 15:**
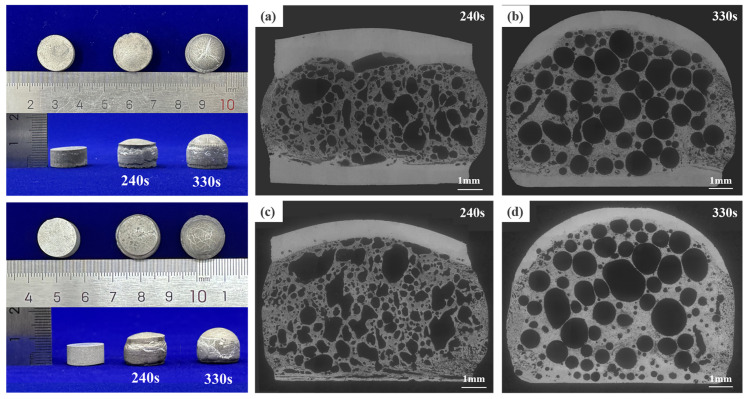
The foam microstructure of AFS precursors was prepared with pins of different thread lengths at 680 °C for different foaming times: (**a**,**b**) 4.00 mm (**c**,**d**) 4.85 mm.

Based on previous studies, the holding temperature for precursor foaming is chosen to be at 680 °C [24]. At 240 s of holding time, the bubbles are produced in the region of the foaming agent contained in the precursor in the action of gases of thermal decomposition of TiH_2_. Because of the short holding time, low amount of decomposition gas, and poor fluidity of the matrix, the resulting bubble holes are not uniform in size, mostly crack-like holes with low roundness, as shown in Figure 15a,c. With the increase of holding time, the matrix gradually softens, while the amount of decomposed gas increases, the bubble holes increase, the roundness rises, and the size is uniform, as shown in Figure 15b,d. In the AFS prepared with a pin thread length of 4.85 mm, there is a panel on one side only at the early stage of foaming, and the other side of the sandwich contains more bubble holes, which will seriously affect the mechanical properties of the AFS, as shown in Figure 15c,d.

**Figure 16:**
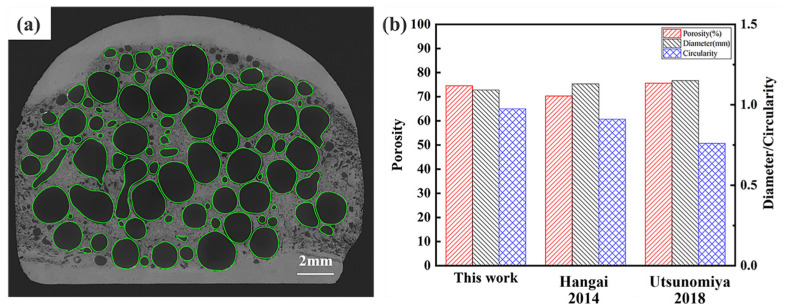
The bubble hole statistics and data comparison: (**a**) schematic of the bubble being counted, (**b**) comparison of bubble porosity, circularity, and diameter. Reprinted with permission from ref. [44]. Copyright 2014 Elsevier. Reprinted with permission from ref. [45]. Copyright 2018 J-Stage.

During the foaming process, cracks are created on the side surfaces of the foamed specimen after expansion. The decomposed gases near the surface partly escape along the cracks, resulting in almost no bubble holes near the side surface of the foamed specimen. Therefore, the regions without bubbles on both sides are not considered in the statistical data, and only the bubbles, as shown in Figure 16a, are counted numerically. The AFS prepared under the process parameters in Table 3 is held at 680 °C for 330 s. The porosity of the foam layer reaches 74.55%, the average pore size is 1.192 mm, and the circularity of the foam pores reaches 0.97. The method of preparing AFS by one welding is able to obtain the same effect as the method of preparing AFS by two welding or opposite placement of precursor foaming, and the roundness of the bubble holes is greatly improved, as shown in Figure 16b.

## 6. Conclusions

In this study, the effects of welding parameters, welding track, and pin thread on the distribution and position of foaming agents in the AFS precursor are investigated by preparing the AFS precursor by FSW. The following conclusions are obtained:

1. The AFS precursors with no defects, uniform distribution of foaming agent, and excellent densification are prepared with a rotation speed of 1500 r/min, travel speed of 25 mm/min, tool plunge depth of 0.2 mm, and multiple passes welding along the RS;
2. The increased welding temperature enhances the softening degree and area of the matrix in the WZ, which improves the area of the AFS precursor prepared by a single FSW and the uniformity of the foaming agent in the precursor, but excessive welding temperatures can result in the occurrence of welding defects;
3. The material in the WZ area flows upward under the action of the pin threads so that AFS precursors with foaming agent distribution in the core layer can be prepared by shortening the length of the threads;
4. The softened area of the matrix is greater on the AS side than on the RS side, and the softened matrix builds up on the RS side as the material flows. The presence of curved layers of unmixed powder and crescent-shaped regions of no foaming agent in the AFS precursors produced along the AS multi-pass FSW.

## Figures and Tables

**Table 1:** Mechanical properties of 7075-O [36].

Density (kg/m^3^)	Tensile Strength (MPa)	Yield Strength (MPa)	Elongation (%)
2700	207	95	21.4

**Table 2:** Main process parameters.

Material	α	A	n	Q	$\mathrm{Densities}/kg\cdot m^{-3}$	$\mathrm{Thermal}\;\mathrm{Conductivity}/W\cdot m^{-1}\cdot K^{-1}$	$\mathrm{Specific}\;\mathrm{Heat}/J\cdot {Kg}^{-1}\cdot K^{-1}$
7075	$1.03\times 10^{9}$	0.0141	5.41	129,000	2810	$74.07+0.25T-4.621\times 10^{-5}T^{2}$	$850.42+1.21T-4.396\times 10^{-4}T^{2}$

**Table 3:** FSW welding process parameters.

Rotation Speed (r/min)	Travel Speeds (mm/min)	Tool Plunge Depth (mm)	Overlap Direction
1500	25	0.2	RS

## Data Availability

The original contributions presented in the study are included in the article, further inquiries can be directed to the corresponding author.
